# Effects of Anthocyanin on Serum Lipids in Dyslipidemia Patients: A Systematic Review and Meta-Analysis

**DOI:** 10.1371/journal.pone.0162089

**Published:** 2016-09-02

**Authors:** Changfeng Liu, Jinfeng Sun, Yan Lu, Yacong Bo

**Affiliations:** 1 The First Affiliated Hospital of Zhengzhou University, 450052 Zhengzhou, Henan, China; 2 Department of Social Medicine and Health Service Management, College of Public Health, Zhengzhou University, 450001 Zhengzhou, PR China; 3 The North Hospital of the Ninth People’s Hospital of Shanghai City, 201999, Shanghai, China; 4 Department of Nutrition and Food Hygiene, College of Public Health, Zhengzhou University, 450001 Zhengzhou, Henan, China; Universita degli Studi di Milano, ITALY

## Abstract

**Background:**

Dyslipidemia was present in most of the patients with coronary heart disease. Epidemiological evidence suggests that anthocyanin has some effects on the serum lipid. However, these results are controversial. This study aimed at collecting current clinical evidence and evaluating the effects of anthocyanin supplementation on total cholesterol (TC), triglyceride (TG), low-density lipoprotein cholesterol (LDL-C), and high-density lipoprotein cholesterol (HDL-C) in dialysis patients.

**Methods:**

The search included PubMed, Web of Science, MEDLINE, Cochrane Library, China National Knowledge Infrastructure, Wanfang Database (up to July 2015) to identify randomized controlled trials (RCTs) on the association between anthocyanin and serum lipids. RevMan (version 5.2) was used for Meta-analysis. Meta-regression analysis, sensitivity analysis and Egger’s weighted regression tests were performed by using STATA software (version 12.0; StatCorp, College Station, TX, USA).

**Results:**

Six studies (seven arms) involving 586 subjects were included in this meta-analysis. The results showed that anthocyanin supplementation has significant effects on TC [MD = -24.06, 95% *CI*(-45.58 to -2.64) mg/dL, *I*^2^ = 93%], TG [MD = -26.14, 95%*CI*(-40.20 to -3.08) mg/dL, *I*^2^ = 66%1], LDL-C [MD = -22.10, 95% *CI* (-34.36 to -9.85) mg/dL, *I*^2^ = 61%], and HDL-C(MD = 5.58, 95% *CI* (1.02 to 10.14) mg/dL;*I*^2^ = 90%).

**Conclusion:**

Anthocyanin supplementation significantly reduces serum TC, TG, and LDL-C levels in patients with dyslipidemia, and increases HDL-C. Further rigorously designed RCTs with larger sample sizes are needed to confirm the effectiveness of anthocyanin supplementation for dyslipidemia, especially hypo high density lipoprotein cholesterolemia.

## 1 Introduction

Mortality and disease burden of cardiovascular disease (CVD) is increasing globally[1]. According to the World Health Organization, an estimated 17.3 million people died of CVDs in 2008, representing 30% of all global deaths[2]. Besides, almost 25 million people worldwide will die of CVDs per year by 2020[3]. Dyslipidemia was present in 90.1% of patients with coronary heart disease[4]. A meta-analysis of randomized controlled trials (RCTs) concluded that a 40% reduction in low-density lipoprotein cholesterol (LDL-C) with a 30% increase in high-density lipoprotein cholesterol (HDL-C) could lower the risk cardiovascular by 70%[5]. To date, several classes of lipid-modulating agents have been introduced: ezetimibe, monoclonal antibody anti-PCSK9bile, acid consolidation agent, inhibitors of 3-hydroxy-3-methylglutaryl coenzyme A (HMG-CoA, for example, statins), and so on, which have considerable benefit for improving serum lipids profile but also have a number of drawbacks. For example, statins are known to give rise to some serious adverse effects such as inflammatory (dermatomyositis/ polymyositis) and necrotizing myopathies[6]. Due to these concerns, there has been an increasing attempts to use functional natural products as alternatives to the conventional lipid-modulating treatments, which are often more acceptable to patients.

Anthocyanin, one of the largest groups of water-soluble red, purple, and blue natural pigments, is the most common class of phenolic compounds, which are largely distributed in the human diet through crops, beans, fruits, vegetables, and red wines[7]. They have a complex structure of an aliphatic or aromatic three-ring molecular area, one or more attached sugar, and sometimes acyl groups attached to sugar, which is very reactive towards reactive oxygen species (ROS) and have been reported to have positive effects on the treatment of various diseases[8]. Anthocyanin was prescribed as medicines in many countries. A large amount of researches suggested that anthocyanin can improve dyslipidemia[9], antioxidant capacity[10], visual function[11], and microcirculation[12], prevent insulin resistance[13], reduce inflammatory reaction[14], and incidences of urinary tract infection inhibit the replication of virus and cell growth of cancer. Its role of improving lipid profile was concerned by researchers. But the findings are contradictory.

Several studies have reported that anthocyanin supplementation reduced TC and TG. However, others failed to show the effects. To the best of our knowledge, this is the first meta-analysis to evaluate the effects of anthocyanin supplementation on lipid profile in patients with dyslipidemia based on RCTs.

## 2 Materials and Methods

The meta-analysis was conducted according to the Preferred Reporting Items for Systematic Reviews and Meta-analyses (PRISMA) Guidelines. Even though the strategy for this review was discussed and agreed, no official protocol was published or registered.

### 2.1 Search strategy

The PubMed, Web of Science, MEDLINE, Cochrane Library, China National Knowledge Infrastructure, and Wanfang Database (up to July 2015) were searched to identify eligible studies. The following combination of search terms were used in databases in both English and Chinese: (anthocyanin OR anthocyanin extract OR cyanidin OR pelargonidin OR delphindin OR peonidin OR petunidin) AND (hyperlipidemia OR dyslipidemia OR hyperlipidemic OR hypolipidemic OR hypercholesterolemic OR hypercholesterolemia OR dyslipidemic OR hypocholesterolemic OR cholesterol OR triglycerides OR hypertriglyceridemia OR hypotriglyceridemic OR ‘‘high-density lipoprotein” OR ‘‘low-density lipoprotein”). Detailed processes of search strategy are shown in S1 Text.

### 2.2 Data selection

Two investigators (Changfeng Liu and Yacong Bo) independently screened the titles and abstracts of the articles to evaluate eligibility for inclusion until consensus was reached. Articles included should meet all the following criteria: (1) the studies should be designed as RCTs; (2) the participants should be dyslipidemic adults aged 18 years or older; (3) the intervention of interest was purified anthocyanin or anthocyanin extract administered; (4) the duration of intervention should be at least 4 weeks; (5) lipid profile (TC, TG, LDL-C, HDL-C) data at baseline and after intervention of anthocyanin were available.

### 2.3 Data extraction

Two authors (Changfeng Liu and Yacong Bo) independently extracted the following data from each eligible study: publication year, administrated daily dose of anthocyanin, study design, duration of treatment period, type of study population, age, means and standard deviations (SDs) of serum lipid profiles at the baseline and at the end of trials, and number of participants. Discrepancies were resolved by a third investigator.

### 2.4 Assessment of risk of bias

Two reviewers (Changfeng Liu and Yacong Bo) independently assessed the risk of bias as recommended by the Cochrane Hand book for Systematic Reviews of Interventions[15]. The following methodological domains were considered: random sequence generation, allocation concealment, blinding of participants and personnel, blinding of outcome assessment, incomplete outcome data, selective reporting, and other potential threats to validity. We explicitly judged each of the domains as having high risk, low risk or unclear risk of bias.

### 2.5 Data analysis

The unit of serum lipid levels was mmol/L which was converted into mg/dL by multiplying 38.67 (TC, HDL-C and LDL-C) or 88.545 (TG) respectively.

The mean difference (MD) for net change and 95% CI was used to determine the effect of anthocyanin supplementation on TC, TG, LDL-C and HDL-C in this meta-analysis[15]. The following formula was used to calculate the Mean difference for the net changes Mean difference = Mean_pre-treatment_—Mean_post-treatment_. And the following formula was used to calculate the standard deviations (SDs) for the net changes: SD = square root [(SD pre-treatment)^2^ + (SD post-treatment)^2^ - (2R × SD pre-treatment × SD post-treatment)], assuming a correlation coefficient (R) = 0.5[16, 17]. Studies were assessed for statistical heterogeneity by using the *χ*^2^ tests and the amount of heterogeneity was estimated using *I*^2^ statistic. A fixed-effect model was used if pooling seemed appropriate in view of clinical and methodological similarities between studies where *I*^2^ was below 50%. While when *I*^2^ was beyond 50%, a randomized-effect model would be adopted[15].

Meta-regression analysis was used to determine potential effect modification of variables including age, BMI, dose of anthocyanin supplementation, intervention duration, sample size, baseline concentration of serum lipids in response to supplementation. Besides, a stratified analysis by country was also performed. In order to evaluate the influence of each study on the overall effect size, sensitivity analysis was conducted using the leave-one-out approach. Potential publication bias was explored by using Begg’s funnel plot and Egger’s weighted regression tests. All statistical analyses were conducted using RevMan (version 5.2) and STATA software (version 12.0; StatCorp, College Station, TX, USA). *P*< 0.05 was considered as statistically significant.

## 3 Results

### 3.1 Description of studies

**Fig 1:**
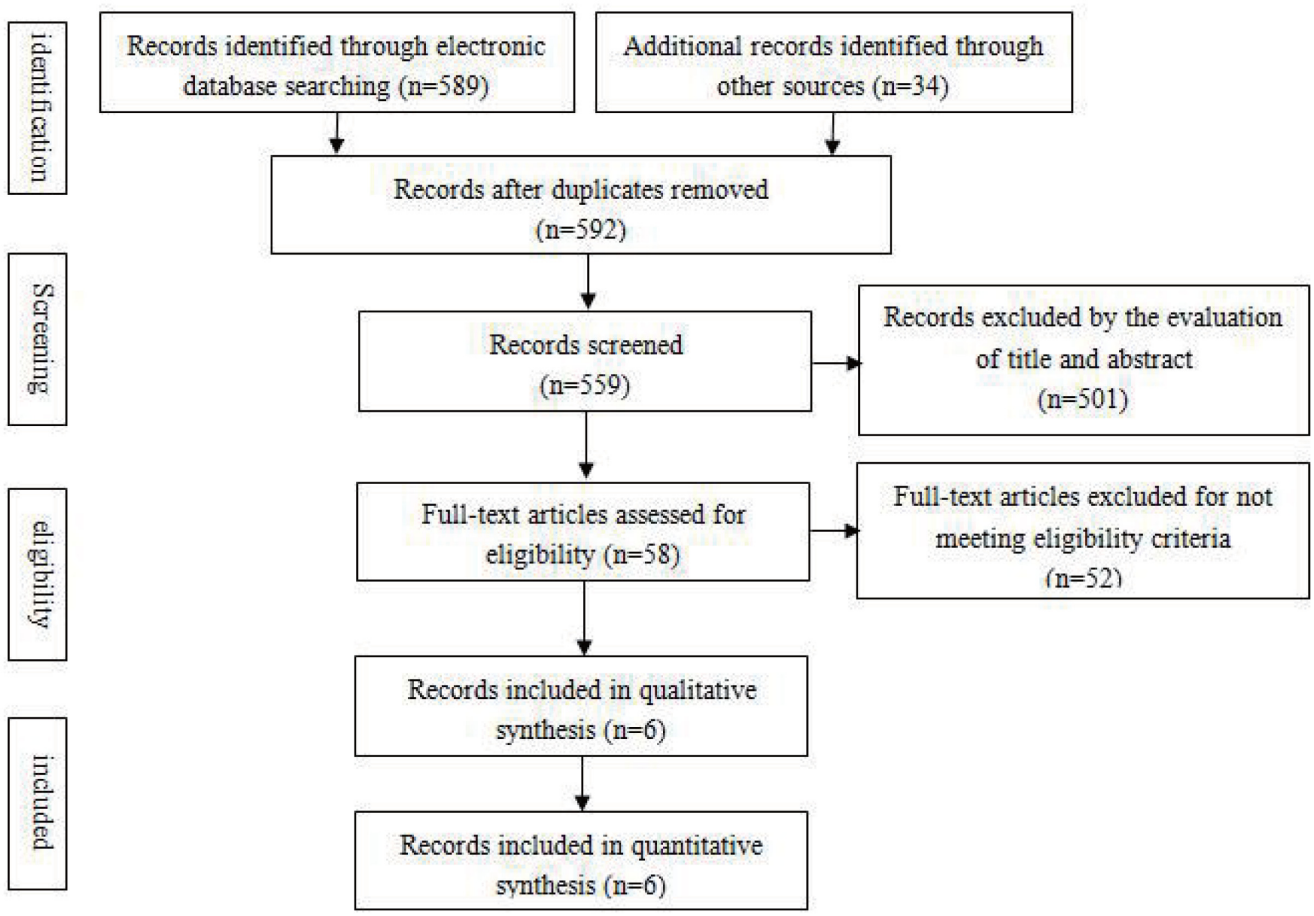
Flow diagram of the study selection process. Flow chart of number of studies identified and included into the review.

As shown in Fig 1, a total of 623 articles were found in the initial search, 559 papers were assessed after removing 64 duplicate articles. Five hundred and one articles were reviewed in full after reviewing the title and abstract. A total of 8 were found to be eligible following the included criteria. Two trials were excluded for having the same population with one included study[18]. At last, six articles were included in the meta-analysis[9, 18–22]. Among them, one study reported data on TG by Geometric mean[18] and one trail did not report LDL-C[20]. Four studies were conducted in China, the other two studies come from Iran. The main characters of these studies are presented in Table 1.

**Table 1:** Demographic characteristics and baseline parameters of included studies in this study.

Study, year		Qin, 2009	Zhu, 2013	Kianbakht, 2013	Soltani, 2014	MU, 2010	QIN, 2008
Location		China	China	Iran	Iran	China	China
Design		R, D, P	R, D, P	R, D, P	R, D, P	R, P	R, P
Duration of trial		12 weeks	24 weeks	2 months	4 weeks	12 weeks	45 days
anthocyanins dose		320mg/d	320 mg/d	259.68 mg/d	90 mg/d	200 mg/d	200 mg/d
Participants	anthocyanins	60	73	40	25	30	51
	Placebo	60	73	40	25	28	51
Age, years	anthocyanins	40–65	40–65	51.3±15.27	48.08±16.39	55.3±5.4	56.16±8.13
	Placebo	40–65	40–65	55.8±13.28	46.36±16.59	55.2±5.3	57.08±8.76
BMI, kg/m^2^ (Mean±SD)	anthocyanins	25.5±3.1	NR	30.3±3.5	25.40±1.75	24.76±2.94	NR
	Placebo	26.7±4.0	NR	29.6±4.3	25.21±2.01	26.34±3.93	NR
TC, mg/dL (Mean±SD)	anthocyanins	226.2±35.5	249.4±39.4	277.9±26.92	226.48±32.09	226.6±35.2	215.8±30.5
	Placebo	224.3±36.4	250.6±32.5	281.75±38.72	220.20±45.76	223.1±30.9	210.8±28.2
TG, mg/dL (Mean±SD)	anthocyanins	197.9±87.0	NR	295.48±24.53	226.20±96.99	187.7±69.1	206.3±91.2
	Placebo	205.8±83.0	NR	304.98±22.84	191.36±56.54	215.2±128.4	209.9±89.3
LDL, mg/dL (Mean±SD)	anthocyanins	159.2±34.4	129.2±22.4	157.05±21.96	132.80±23.76	163.9±32.5	NR
	Placebo	158.5±37.8	127.2±18.2	172.45±48.73	121.0 ±32.06	158.2±37.9	NR
HDL, mg/dL (Mean±SD)	anthocyanins	45.9±8.5	47.2±8.9	45.68±4.72	45.76±9.73	45.2±8.5	46.4±10.8
	Placebo	46.1±9.6	47.9±8.1	43.68±4.97	46.56±10.52	46.0±9.3	49.5±9.3

*Note*: R, randomized; D, double blind; P, parallel trial; NR, not reported; BMI, body mass index

### 3.2 Risk of bias in included studies

**Fig 2:**
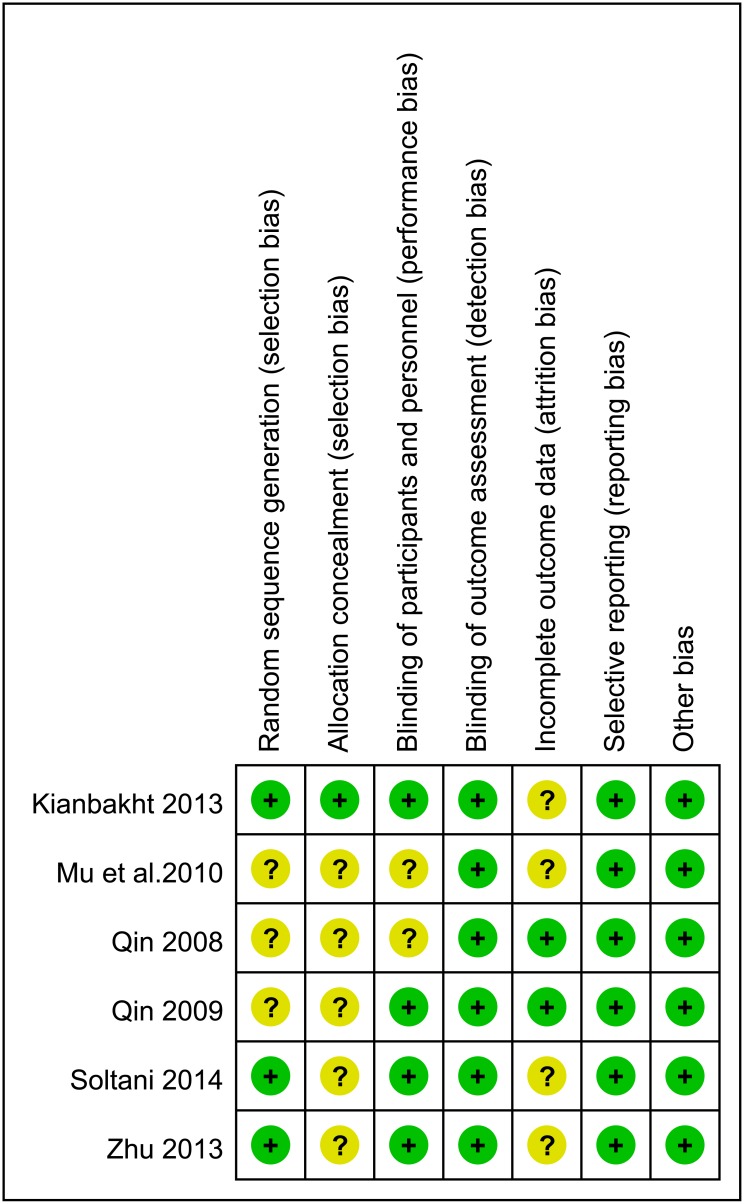
Risk of bias assessment of included studies.

The assessment of risk of bias was presented in the ‘Risk of bias assessment of included studies’ (Fig 2). All trials were described as randomized trial design, while three of six studies did not show the detail information about random sequence generation[9, 20–22], and five trials did not describe the methods of allocation concealment[9, 18–20, 22]. Blinding of participants and personnel (performance bias) were considered to be unclear in two trials[20, 22].

In order to judge the risk of bias for incomplete outcome data (attrition bias), we considered whether intention-to-treat (ITT) analysis was conducted for primary outcomes. None of the six trials carried out ITT analysis. Among them, the dropout rates for 3 trails ranged from 2.2% to 7.41%, while one trail was 23.81%, which may affect the estimation[21]. The other five trials have almost equal number of dropout from each intervention group. We focused on baseline comparability for other potential sources of bias. In all trials the intervention and control groups were reported to be comparable at baseline for the lipid levels.

### 3.3 Effects of interventions

**Fig 3:**
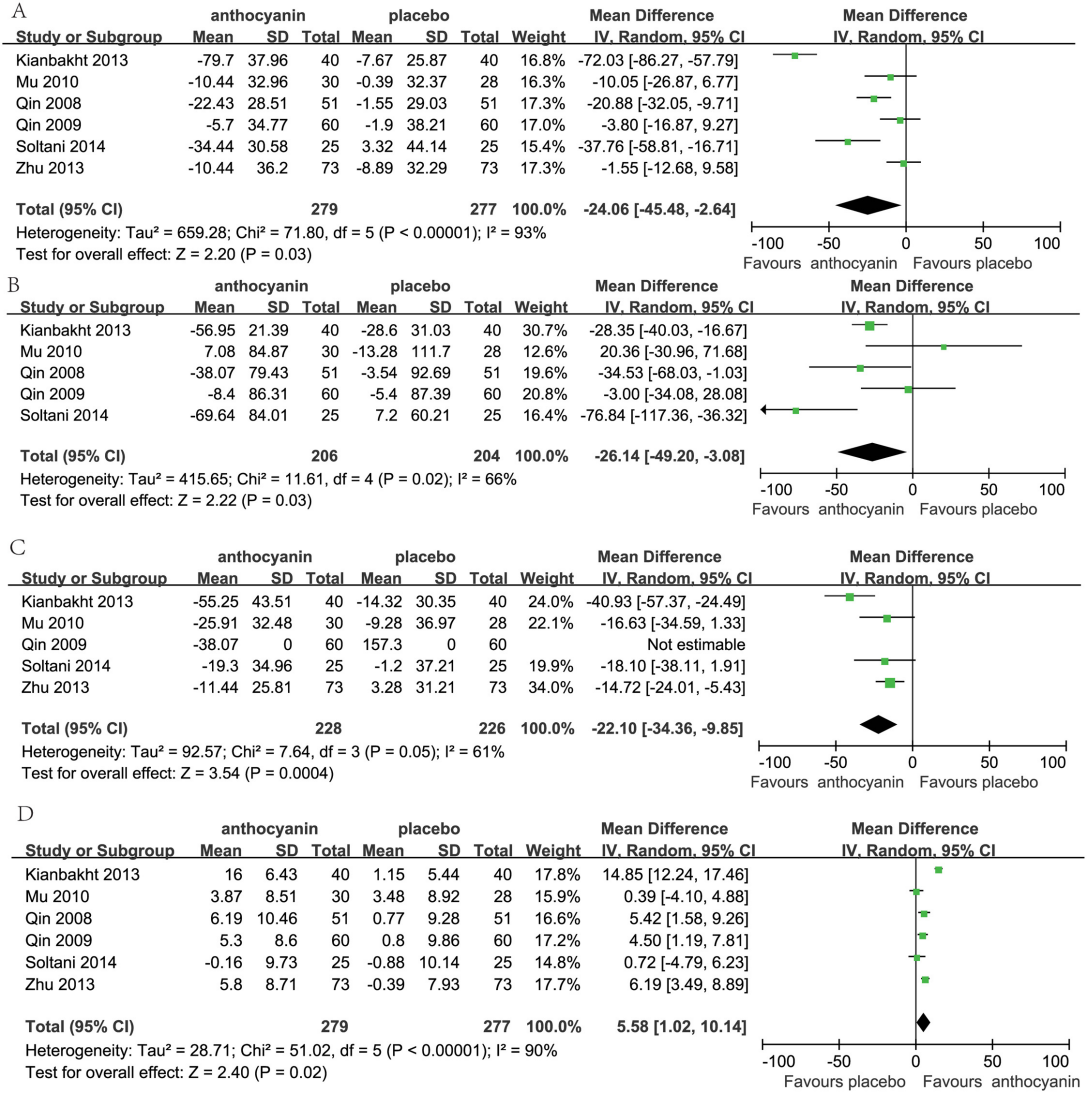
Forest plot between anthocyanin supplementation and serum lipids (A: total cholesterol, B: triglycerides, C: low-density lipoprotein cholesterol, D: high-density lipoprotein cholesterol).

The effects of anthocyanin supplementation on the levels of TC, TG, LDL-C and HDL-C in all studies are shown in Fig 3. Overall, the pooled analysis showed that the anthocyanin supplementation group had lower levels of TC [MD = -24.06, 95% *CI*(-45.58 to -2.64) mg/dL, *I*^2^ = 93%] (Fig 3A), TG [MD = -26.14, 95%*CI*(-49.20 to -3.08) mg/dL, *I*^2^ = 66%1] (Fig 3B), LDL-C [MD = -22.10, 95% *CI* (-34.36 to -9.85) mg/dL, *I*^2^ = 61%](Fig 3C) than the placebo group. However, anthocyanin supplementation can significantly increase the level of HDL-C [MD = 5.58, 95% *CI* (1.02 to 10.14) mg/dL; *I*^2^ = 90%] (Fig 3D) compared with placebo group. When we carried out the stratified analysis by country, significant results of TC were observed among both Iranian population (MD = -50.58, 95% *CI*(-86.52 to -14.64) mg/dL, *I*^2^ = 89%) and Chinese population (MD = -6.59, 95% *CI*(-12.44 to -0.73) mg/dL, *I*^2^ = 1%).

### 3.4 Sensitivity and heterogeneity analysis

Results of the sensitivity analysis showed that the observed lack of difference for any of the evaluated lipid parameters could not be attributed to a single study. There was a significant heterogeneity for the impact of anthocyanin supplementation on serum lipids levels. Meta-regression with age, BMI, dose of anthocyanin supplementation, intervention duration, sample size, baseline concentration of serum lipids, and country showed no significant impact on between-study heterogeneity (P>0.05). The leave-one-out analysis showed that the key contributor to this high heterogeneity was one study conducted by Kianbakht et al[21]. After excluding it, the heterogeneity was reduced to I^2^ = 70% for TC, I^2^ = 0% for LDL-C, and I^2^ = 41% for HDL-C. But significances of the pooled changes were not altered, which demonstrated that the results were robust.

### 3.5 Publication bias

Egger’s regression test and funnel plots were used to detect the potential publication bias. Egger test showed no evidence of significant publication bias for the effects of anthocyanin supplementation on each parameter: including TC (t = -2.98, P = 0.059), TG (t = -2.60, P = 0.122), LDL-C (t = -0.27, P = 0.813), and HDL-C (t = 0.84, P = 0.461). The funnel plots were provided in S1 Fig.

## 4 Discussion

Our meta-analysis showed supplementation with anthocyanin was associated with a decrease in TC, TG, and LDL-C, but an increased effect on HDL-C compared with controls. Funnel plots and Egger’s regression test showed no publication bias for all the parameters. To our knowledge, the present study is the first meta-analysis to explore the association between anthocyanin supplementation and serum lipid based on RCTS.

Epidemiological studies suggested that the consumption of anthocyanin-rich foods and beverages has vaso protective effects in human. A growing body of studies suggests that oxidative stress is thought to play a pivotal role in the pathogenesis of a number of chronic inflammatory disease processes including atherosclerosis. However, it remains unclear what their mechanism of action is. Several potential mechanisms might explain the inverse association between anthocyanin supplementation and TC, TG, LDL-C, and HDL-C. Firstly, anthocyanins reduced plasma TC possibly mediated by increasing fecal excretion of both neutral sterols and acidic, moreover, anthocyanin could down-regulate the gene expression of hepatic HMG-CoA reductase, which inhibited the synthesis of cholesterol [23]. Secondly, the TG-lowering effect of anthocyanin may be ascribed to the reductions in serum apo B–and apo C-III–containing TG rich particles[13]. Thirdly, anthocyanin supplementation in dyslipidemic patients had a beneficial effect on the decreasing in LDL-C concentrations, which may be partially mediated via the inhibition of cholesteryl ester transfer protein (CEPT)[9], a plasma protein that mediates the removal of cholesteryl esters from HDL in exchange for a TG molecule derived primarily from either LDL, VLDL, or chylomicrons[24]. In addition, anthocyanin increased the expression of LDL-receptor and cholesterol excretion in feces [25], which resulted in an improved clearance of plasma LDL-C.

There was a significant heterogeneity for the impact of anthocyanin supplementation on serum lipids levels. The leave-one-out analysis showed that the key contributor to this high heterogeneity was one study conducted by Kianbakht et al[21]. After excluding it, the heterogeneity was reduced to I^2^ = 70% for TC, I^2^ = 0% for LDL-C, and I^2^ = 41% for HDL-C. By comparing these six studies, we found that follow-up rate for this study is only 80%, whereas the follow-up rate of the other studies are all more than 90%. Therefore, we infer that the follow-up rate might be partly responsible for the heterogeneity.

Several limitations should be acknowledged. Firstly, the present meta-analysis focused only on papers published in Chinese and English, the ones that reported in other languages may increase heterogeneity in the present results. Secondly, the subjects included in this study came from China and Iran. Due to this limitation, the results are applicable to the population in these two countries, but cannot be extended to populations elsewhere. Thirdly, due to the limitation of sample size, we could not conduct subgroup analyses. Further rigorously designed RCTs with larger sample sizes are needed to confirm the effectiveness of anthocyanin supplementation for dyslipidemia.

## 5 Conclusion

The present meta-analysis demonstrated that anthocyanin supplementation significantly reduces serum TC, TG, and LDL-C levels in patients with dyslipidemia, but an increased effect on HDL-C compared with controls. Further rigorously designed RCTs with larger sample sizes are needed to confirm the effectiveness of anthocyanin supplementation for dyslipidemia, especially hypo high density lipoprotein cholesterolemia.

## Supporting Information

S1 Fig Funnel plot for publication bias of anthocyanin supplementation and serum lipids (A: total cholesterol, B: triglycerides, C: low-density lipoprotein cholesterol, D: high-density lipoprotein cholesterol). (EPS)

S1 Table PRISMA 2009 checklist. (DOC)

S1 Text Search strategy. (DOCX)

## References

[1] Yusuf S, Reddy S, Ounpuu S, Anand S. Global burden of cardiovascular diseases—Part I: General considerations, the epidemiologic transition, risk factors, and impact of urbanization. Circulation. 2001;104(22):2746–53. 10.1161/hc4601.099487. WOS:000172432500042. 11723030

[2] Johnson SA, Figueroa A, Navaei N, Wong A, Kalfon R, Ormsbee LT, et al Daily Blueberry Consumption Improves Blood Pressure and Arterial Stiffness in Postmenopausal Women with Pre- and Stage 1-Hypertension: A Randomized, Double-Blind, Placebo-Controlled Clinical Trial. Journal of the Academy of Nutrition and Dietetics. 2015;115(3):369–77. 10.1016/j.jand.2014.11.001. WOS:000350231700007. 25578927

[3] Bhatnagar P, Wickramasinghe K, Williams J, Rayner M, Townsend N. The epidemiology of cardiovascular disease in the UK 2014. Heart. 2015;101(15):1182–9. 10.1136/heartjnl-2015-307516. WOS:000357729600006. 26041770 PMC4515998

[4] Rinkuniene E, Petrulioniene Z, Laucevicius A, Ringailaite E, Laucyte A. Prevalence of conventional risk factors in patients with coronary heart disease. Medicina-Lithuania. 2009;45(2):140–6. WOS:000264691100007. 19289904

[5] Abdel-Maksoud M, Sazonov V, Gutkin SW, Hokanson JE. Effects of modifying triglycerides and triglyceride-rich lipoproteins on cardiovascular outcomes. Journal of Cardiovascular Pharmacology. 2008;51(4):331–51. 10.1097/FJC.0b013e318165e2e7. WOS:000255492200001. 18427276

[6] Padala S, Thompson PD. Statins as a possible cause of inflammatory and necrotizing myopathies. Atherosclerosis. 2012;222(1):15–21. 10.1016/j.atherosclerosis.2011.11.005. WOS:000302960600003. 22154355

[7] Afford S, Randhawa S. Apoptosis. Molecular Pathology. 2000;53(2):55–63. 10.1136/mp.53.2.55 10889903 PMC1186906

[8] Cao GH, Muccitelli HU, Sanchez-Moreno C, Prior RL. Anthocyanins are absorbed in glycated forms in elderly women: a pharmacokinetic study. American Journal of Clinical Nutrition. 2001;73(5):920–6. WOS:000168359900012. 11333846 10.1093/ajcn/73.5.920

[9] Qin Y, Xia M, Ma J, Hao Y, Liu J, Mou H, et al Anthocyanin supplementation improves serum LDL- and HDL-cholesterol concentrations associated with the inhibition of cholesteryl ester transfer protein in dyslipidemic subjects. American Journal of Clinical Nutrition. 2009;90(3):485–92. 10.3945/ajcn.2009.27814. WOS:000269257300006. 19640950

[10] Wang Y, Zhao L, Wang D, Huo Y, Ji B. Anthocyanin-rich extracts from blackberry, wild blueberry, strawberry, and chokeberry: antioxidant activity and inhibitory effect on oleic acid-induced hepatic steatosis in vitro. Journal of the Science of Food and Agriculture. 2015:n/a–n/a. 10.1002/jsfa.7370 26250597

[11] Shim SH, Kim JM, Choi CY, Kim CY, Park KH. Ginkgo biloba Extract and Bilberry Anthocyanins Improve Visual Function in Patients with Normal Tension Glaucoma. Journal of Medicinal Food. 2012;15(9):818–23. 10.1089/jmf.2012.2241. WOS:000308365900008. 22870951 PMC3429325

[12] No authors listed. The role of whortleberry anthocyanosides in the microcirculation. Minerva chirurgica. 1993;48(17):XIII–XIII. MEDLINE:8290121. 8290121

[13] Li D, Zhang Y, Liu Y, Sun R, Xia M. Purified Anthocyanin Supplementation Reduces Dyslipidemia, Enhances Antioxidant Capacity, and Prevents Insulin Resistance in Diabetic Patients. Journal of Nutrition. 2015;145(4):742–8. 10.3945/jn.114.205674. WOS:000352180500011. 25833778

[14] Hassellund SS, Flaa A, Kjeldsen SE, Seljeflot I, Karlsen A, Erlund I, et al Effects of anthocyanins on cardiovascular risk factors and inflammation in pre-hypertensive men: a double-blind randomized placebo-controlled crossover study. Journal of Human Hypertension. 2013;27(2):100–6. 10.1038/jhh.2012.4. WOS:000317116800007. 22336903

[15] GSe HJ. Cochrane Handbook for Systematic Reviews of Interventions Version 5.1.0. Available: www.cochrane-handbook.org: The Cochrane Collaboration; 2011.

[16] Hozo SP, Djulbegovic B, Hozo I. Estimating the mean and variance from the median, range, and the size of a sample. BMC medical research methodology. 2005;5:13-. 10.1186/1471-2288-5-13. MEDLINE:15840177. 15840177 PMC1097734

[17] Higgins J, Thompson S, Deeks J, Altman D. Statistical heterogeneity in systematic reviews of clinical trials: a critical appraisal of guidelines and practice. Journal of health services research & policy. 2002;7(1):51–61. 10.1258/1355819021927674. MEDLINE:11822262. 11822262

[18] Zhu Y, Ling W, Guo H, Song F, Ye Q, Zou T, et al Anti-inflammatory effect of purified dietary anthocyanin in adults with hypercholesterolemia: A randomized controlled trial. Nutrition Metabolism and Cardiovascular Diseases. 2013;23(9):843–9. 10.1016/j.numecd.2012.06.005. WOS:000324538200007. 22906565

[19] Soltani R, Hakimi M, Asgary S, Ghanadian SM, Keshvari M, Sarrafzadegan N. Evaluation of the Effects of Vaccinium arctostaphylos L. Fruit Extract on Serum Lipids and hs-CRP Levels and Oxidative Stress in Adult Patients with Hyperlipidemia: A Randomized, Double-Blind, Placebo-Controlled Clinical Trial. Evidence-Based Complementary and Alternative Medicine. 2014 10.1155/2014/217451. WOS:000330675100001. PMC3920853 24587807

[20] Yu QIN, Wenhua L. Effect of Anthocyanin-rich Exact from Black Rice on Patients with Hhyperlipidemia. Food Science. 2008;29(10):540–2. CSCD:3463934.

[21] Kianbakht S, Abasi B, Dabaghian FH. Improved Lipid Profile in Hyperlipidemic Patients Taking Vaccinium arctostaphylos Fruit Hydroalcoholic Extract: A Randomized Double-Blind Placebo-Controlled Clinical Trial. Phytotherapy Research. 2014;28(3):432–6. 10.1002/ptr.5011. WOS:000332988100013. 23686894

[22] Mu H, Qu Q, Liu J, Qin Y, Ling W, Ma J. EFFECT OF ANTHOCYANINS ON OXIDATIVE STRESS IN SUBJECTS WITH HYPERLIPIDEMIA. Acta Nutrimenta Sinica. 2010;32(6):551–5. CSCD:4102476.

[23] Liang Y, Chen J, Zuo Y, Ma KY, Jiang Y, Huang Y, et al Blueberry anthocyanins at doses of 0.5 and 1% lowered plasma cholesterol by increasing fecal excretion of acidic and neutral sterols in hamsters fed a cholesterol-enriched diet. European Journal of Nutrition. 2013;52(3):869–75. 10.1007/s00394-012-0393-6. WOS:000317078900002. 22684634

[24] Inazu A, Brown ML, Hesler CB, Agellon LB, Koizumi J, Takata K, et al Increased high-density lipoprotein levels caused by a common cholesteryl-ester transfer protein gene mutation. The New England journal of medicine. 1990;323(18):1234–8. 10.1056/nejm199011013231803. MEDLINE:2215607. 2215607

[25] de Souza MO, Souza e Silva L, de Brito Magalhaes CL, de Figueiredo BB, Costa DC, Silva ME, et al The hypocholesterolemic activity of acai (Euterpe oleracea Mart.) is mediated by the enhanced expression of the ATP-binding cassette, subfamily G transporters 5 and 8 and low-density lipoprotein receptor genes in the rat. Nutrition Research. 2012;32(12):976–84. 10.1016/j.nutres.2012.10.001. WOS:000314481600009. 23244543

