# Supplementary material for: Effects of Anthocyanin on Serum Lipids in Dyslipidemia Patients: A Systematic Review and Meta-Analysis
Source: PLoS One. 2016 Sep 2;11(9):e0162089. doi: 10.1371/journal.pone.0162089 (PMC5010219; doi:10.1371/journal.pone.0162089)
Supplement: S1 Text — (DOCX) [file pone.0162089.s003.docx]

# Search strategy

## Search Terms

Searches were conducted in PubMed, Web of Science, MEDLINE, Cochrane Library, [China](javascript:void(0);) [National](javascript:void(0);) [Knowledge](javascript:void(0);) [Infrastructure](javascript:void(0);), and [Wanfang](javascript:void(0);) [Database](javascript:void(0);) for records from inception until March 2015. For all searches we used both Medical Subject Headings (MeSH terms) and text words in PubMed, and Emtree terms and text words in Web of Science, MEDLINE, Cochrane Library, [China](javascript:void(0);) [National](javascript:void(0);) [Knowledge](javascript:void(0);) [Infrastructure](javascript:void(0);). PubMed Search terms were as follows:

## Participants

((“hyperlipidemia” [MeSH Terms] OR “hyperlipidemia” [All Fields]) OR (“dyslipidemia” [MeSH Terms] OR “dyslipidemia” [All Fields]) OR (“hyperlipidemic” [MeSH Terms] OR “hyperlipidemic” [All Fields]) OR (“hypolipidemic” [MeSH Terms] OR “hypolipidemic” [All Fields]) OR (“hypercholesterolemic” [MeSH Terms] OR “hypercholesterolemic” [All Fields]) OR (“hypercholesterolemia” [MeSH Terms] OR “hypercholesterolemia” [All Fields]) OR (“dyslipidemic” [MeSH Terms] OR “dyslipidemic” [All Fields]) OR (“hypocholesterolemic” [MeSH Terms] OR “hypocholesterolemic” [All Fields]) OR (“hypertriglyceridemia” [MeSH Terms] OR “hypertriglyceridemia” [All Fields]) OR (“hypotriglyceridemic” [MeSH Terms] OR “hypotriglyceridemic” [All Fields]) OR (“high-density lipoprotein” [MeSH Terms] OR “high-density lipoprotein” [All Fields]) OR (“low-density lipoprotein” [MeSH Terms] OR “low-density lipoprotein” [All Fields]))

## Intervention

((“anthocyanin” [MeSH Terms] OR “anthocyanin” [All Fields]) OR (“anthocyanin extract” [MeSH Terms] OR “anthocyanin extract” [All Fields]) OR (“cyanidin” [MeSH Terms] OR “cyanidin” [All Fields]) OR (“pelargonidin” [MeSH Terms] OR “pelargonidin” [All Fields]) OR(“delphindin” [MeSH Terms] OR “delphindin” [All Fields]) OR (“peonidin” [MeSH Terms] OR “peonidin” [All Fields]) (“petunidin” [MeSH Terms] OR “petunidin” [All Fields])).
